# A Rare Association: Non-ST Elevation Myocardial Infarction (NSTEMI) Secondary to Respiratory Syncytial Virus (RSV) Infection

**DOI:** 10.7759/cureus.47518

**Published:** 2023-10-23

**Authors:** Jaswanth R Jasti, Hammad S Chaudhry, Sunia S Chaudhary, Narsimha R Jasti

**Affiliations:** 1 Internal Medicine, University of South Dakota Sanford School of Medicine, Sioux Falls, USA

**Keywords:** non-st elevation myocardial infarction (nstemi), acute coronary syndrome, viral infection, coronary artery disease, respiratory syncytial virus (rsv)

## Abstract

We present a case report on a rare association between non-ST elevation myocardial infarction (NSTEMI) and respiratory syncytial virus (RSV) infection in a patient with no traditional risk factors for cardiovascular disease (CVD) including a family history of premature coronary artery disease (CAD). While RSV is commonly known for its respiratory manifestations, it has been increasingly recognized as a cause of significant morbidity and mortality in adults, particularly those with underlying comorbidities. However, the association between RSV infection and NSTEMI, especially in patients without traditional risk factors, remains relatively unexplored. Our case involves a 31-year-old healthy adult who presented with progressive exertional chest pain and flu-like symptoms. Electrocardiogram (EKG) changes and elevated troponin levels indicated NSTEMI. Laboratory tests confirmed RSV infection. Angiography revealed significant coronary artery disease requiring percutaneous coronary intervention. This case highlights the need for healthcare professionals to be aware of the potential cardiovascular (CV) complications associated with RSV infection, even in patients without traditional risk factors. It expands our understanding of viral respiratory infections as potential triggers for acute coronary syndromes (ACS) and emphasizes the importance of considering RSV infection in the differential diagnosis of NSTEMI, especially in young otherwise healthy individuals. Further research is warranted to explore the underlying mechanisms and develop preventive strategies for RSV-related cardiovascular complications.

## Introduction

Respiratory syncytial virus (RSV) from the Paramyxoviridae family is a common respiratory infection that can infect people of all ages. The virus is widespread among children; almost all are infected by two years of age. The clinical manifestations of RSV infection vary with age: while infants and young children are more likely to develop lower respiratory tract infections such as bronchiolitis or pneumonia, most adults with symptomatic RSV infection present with nonspecific upper respiratory symptoms such as rhinorrhea, sore throat, and low-grade fever [1]. Once thought to be primarily a pediatric concern, RSV is increasingly recognized as a cause of significant morbidity and mortality in adults [2]. Growing data now suggests RSV to be an important cause of lower respiratory tract infection in older individuals and those with underlying comorbidities [1]. Of particular concern are the cardiovascular (CV) effects of RSV infection. Previously, many studies that investigated the link between viral respiratory infections and cardiovascular disease (CVD) were primarily focused on influenza [3,4]. More recently, the coronavirus disease 2019 (COVID-19) pandemic has drawn attention to the virus's potential cardiovascular effects, which has increased our understanding of and interest in the pathophysiology of viral respiratory infections [5,6]. However, evidence suggests that RSV, among other viruses, is a significant pathogen in cardiovascular injury, with damage ranging from pericarditis to coronary inflammation and myocardial infarctions [7,8]. In this context, we present a rare case of RSV-induced non-ST elevation myocardial infarction (NSTEMI) in a young healthy adult. We also briefly discuss the possible pathophysiological mechanisms of this uncommon association, as well as the need for greater awareness of the cardiovascular complications of RSV infection.

## Case presentation

**Figure 1:**
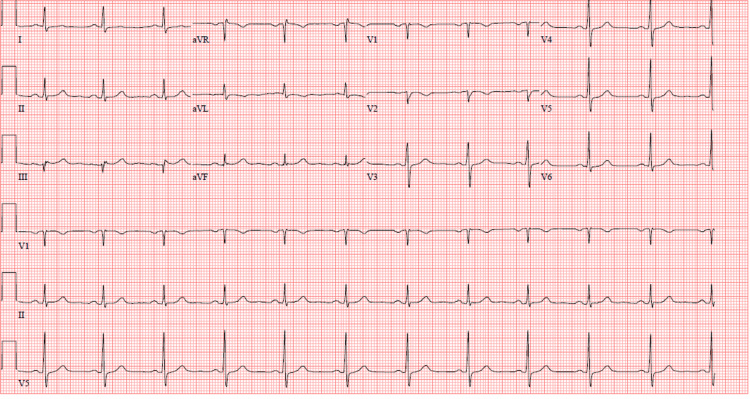
EKG on presentation showing a sinus rhythm, with ST-T wave changes in lateral leads. EKG: electrocardiogram

A 31-year-old male with no significant medical history presented to our hospital complaining of progressive exertional chest pain that started while on a hunting trip. The pain was substernal and pressure-like, radiating to the left arm and neck. The patient stated that he could not play with his children because of the pain, which was also associated with dyspnea and prompted his presentation to the hospital. He was hemodynamically stable on room air with a normal physical examination and without chest wall tenderness. A review of systems revealed that his son had flu-like symptoms about 10 days ago and that the patient had rhinorrhea, throat irritation, a mild cough, and hyperemic eyes for the previous five days. The patient was a nonsmoker, drank alcohol occasionally, and strictly denied using other recreational drugs. He also denied any history of similar complaints or any family history of heart disease. Electrocardiogram (EKG) showed a normal sinus rhythm with mild ST-T wave changes in the lateral leads (Figure 1). Troponin I was elevated at 0.268 ng/mL (normal: 0.00-0.033 ng/mL). Due to concern for acute coronary syndrome (ACS), the patient was loaded with 325 mg of aspirin, and intravenous morphine was given for pain relief.

**Figure 2:**
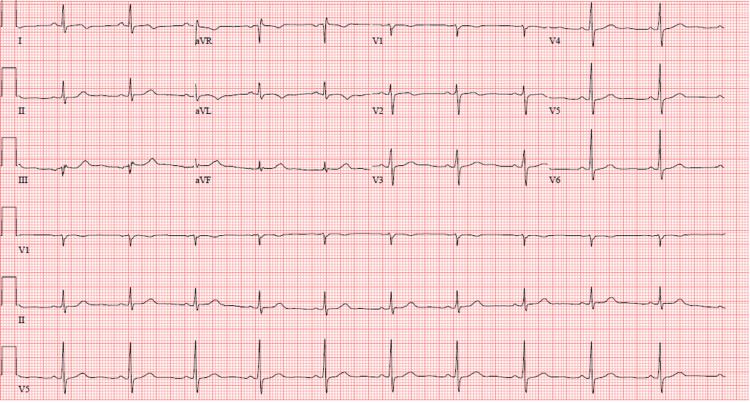
Repeat EKG three hours later showing more pronounced ST-T wave changes in lateral leads. EKG: electrocardiogram

**Figure 3:**
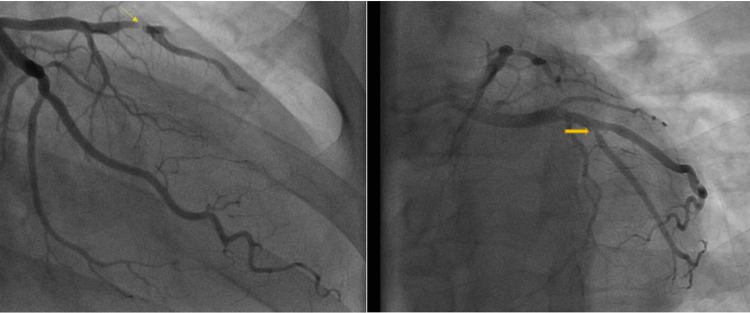
Left heart catheterization showing mid LAD stenosis (yellow arrow on the left) and ostial stenosis of the first diagonal (orange arrow on the right). LAD: anterior descending artery

As the patient was also experiencing flu-like symptoms, a comprehensive respiratory viral panel that included COVID-19 and influenza was obtained and tested positive for RSV. Except for a mild leukocytosis with a white blood cell (WBC) count of 13,000/uL (normal range: 4,000-11,000/uL), all laboratory results, including the complete blood count (CBC), comprehensive metabolic panel (CMP), thyroid-stimulating hormone (TSH), and lipid panel, were within normal limits. Chest X-ray showed clear lungs with a normal-sized heart and no acute cardiopulmonary findings. A three-hour follow-up EKG revealed more pronounced ST-T wave changes in the lateral leads (Figure 2). Subsequent troponins were also increasing, peaking at 0.818 ng/mL. The patient was diagnosed with non-ST elevation myocardial infarction (NSTEMI) based on the nature of his chest pain, rising troponin levels, and changes in the EKG. He was given 300 mg of clopidogrel, started on heparin infusion, and eventually taken for a left heart catheterization. A transthoracic echocardiogram revealed an ejection fraction of 50%-55%, normal cardiac valves, and no regional wall motion abnormalities. Angiogram showed severe coronary artery disease (CAD) with 100% stenosis of the mid left anterior descending artery (LAD), 90% stenosis of distal LAD (LAD lesion had thrombolysis in myocardial infarction {TIMI} 0 flow), and ostial lesion of the first diagonal lesion with 99% stenosis (Figure 3). The patient had successful complex angioplasty with two drug-eluting stents (DES): 1) Y stenting of mid LAD and first diagonal and 2) distal LAD lesion. The patient was admitted to the hospital for 24 hours and discharged with close monitoring from cardiology services the next day.

## Discussion

Cardiovascular disease (CVD) remains the leading cause of death worldwide. In 2020, approximately 19.1 million deaths were attributed to CVD globally [9]. Coronary artery disease (CAD) accounts for most of these deaths and includes stable ischemic heart disease and acute coronary syndromes (unstable angina, NSTEMI, and ST elevation myocardial infarction {STEMI}) [10]. Over the years, the understanding of risk factors for CAD has evolved. While traditional risk factors such as smoking, high blood pressure, high cholesterol, and family history are still important, nontraditional factors such as viral respiratory infections are increasingly recognized as significant contributors. Preceding viral respiratory infection has been reported to predispose to myocardial injury for many years. Some of the most commonly reported cardiac complications of several viral respiratory pathogens, including influenza, parainfluenza, adenovirus, and, more recently, COVID-19, include pericarditis, myocarditis, arrhythmias, and heart failure exacerbations [7]. The precise mechanisms underlying these complications are unknown, but they are believed to involve both direct viral injury and the host immune response. In addition, studies have linked viral respiratory infections, particularly influenza and COVID-19, to acute coronary syndromes (ACS) [3-6]. The pathophysiology of these viral-associated ACS is also poorly understood. Viral infections can activate the immune system, causing systemic inflammation and subsequent destabilization of preexisting coronary artery plaques. Alternatively, the virus can directly damage the coronary arteries' endothelial cells, causing inflammation, platelet activation, and thrombosis [1,2,7].

While much of the research has focused on influenza and COVID-19, RSV has also been linked to cardiovascular complications [7]. Although most known cardiovascular complications of RSV, such as pericarditis, myocarditis, and arrhythmias, have primarily been reported in children, there have been studies of this association in adults [7,11,12]. Since the 1990s, the majority of these have been observational studies, with the most commonly reported complications being exacerbations of underlying heart failure [1,2,8,13]. A case-control study by Guan et al. in 2010 showed that patients with first-time acute myocardial infarction were more likely than control subjects to have positive serum IgG antibodies to RSV, cytomegalovirus (CMV), herpes, adenovirus, and chlamydia [14]. Anderson et al. reported cardiovascular complications (heart failure, atrial fibrillation, and acute coronary events) in both immunocompetent adults (18-65 years of age) and the elderly (>65 years of age) [13]. In addition to the cardiovascular complications demonstrated in previous studies of laboratory-confirmed RSV, studies of influenza-like illness (ILI), summarized in a systematic review [3] and two meta-analyses [15,16], report additional indirect evidence for RSV-associated cardiac complications. While more research into the pathophysiology of RSV's cardiovascular effects would be beneficial, there is evidence that it may share some of the mechanisms described above with other viral infections.

Palivizumab, a humanized monoclonal antibody to the RSV fusion (F) glycoprotein shown to reduce hospitalizations in children, has not been studied in adults [17]. Treating RSV infection in adults is mainly supportive and focused on possible complications.

In our case, the patient presented with acute chest pain, dyspnea, and a recent history of flu-like symptoms. Ischemic changes on the EKG and elevated troponin levels suggest acute coronary syndrome. While the patient had no traditional risk factors for CAD, angiography revealed significant coronary artery disease in the context of laboratory-confirmed RSV infection. There was no evidence of RSV infection involving the lower respiratory tract in the patient. He is also otherwise healthy, with no known chronic autoimmune processes that could have contributed to ACS, making RSV the most likely cause. He underwent standard treatment for NSTEMI, including percutaneous coronary intervention and stenting of the affected vessels, followed by secondary prevention.

## Conclusions

In addition to traditional risk factors, viral respiratory infections, such as RSV, should be considered a risk factor for CAD. More research into the CV effects of RSV infection in adults is needed. Because there are no specific treatment options for RSV infection in adults, the emphasis should be on prevention, including vaccination: in May 2023, the US Food and Drug Administration approved Arexvy, the first vaccine for the prevention of lower respiratory tract disease caused by RSV in individuals 60 years of age and older.
